# Somatic Embryogenesis and Genetic Transformation of *Caragana intermedia*

**DOI:** 10.3390/plants14101545

**Published:** 2025-05-21

**Authors:** Ju Tian, Jialei Zhu, Xiaohan Deng, Xu Zhu, Ruigang Wang, Guojing Li

**Affiliations:** 1 Inner Mongolia Key Laboratory of Plants Adversity Adaptation and Genetic Improvement in Cold and Arid Regions, Inner Mongolia Agricultural University, Hohhot 010018, China; tianju99@163.com (J.T.); dengxiaohan394@163.com (X.D.); zzsgptyy@163.com (X.Z.); wangruigang@imau.edu.cn (R.W.); 2 School of Biology and Agriculture, Shaoguan University, Shaoguan 512005, China; zhujialei99@163.com

**Keywords:** cotyledon, plant tissue culture, embryogenic callus, morphogenesis, GMO

## Abstract

*Caragana intermedia* is a perennial shrub species in the genus *Caragana* (Fabaceae), demonstrating remarkable stress resistance and adaptability. However, research on its somatic embryogenesis (SE) and genetic transformation techniques remains limited. In this study, we established an SE system by utilizing immature cotyledons isolated from young *C. intermedia* seeds. Our findings demonstrated that the immature cotyledons at 6–7 weeks after flowering (WAF) were the best explants for SE. The optimal embryo induction medium consisted of an MS basal medium supplemented with 5 mg/L α-naphthaleneacetic acid (NAA), 3 mg/L 6-benzylaminopurine (6-BA), 30 g/L sucrose, 7 g/L agar, and 500 mg/L hydrolyzed casein. Cotyledon-stage embryos germinated on a half-strength MS medium, exhibiting a 34.36% germination rate. Based on the SE system, we developed a preliminary genetic transformation system using the *RUBY* reporter gene, which successfully generated transgenic calli and cotyledon-stage embryos. The establishment of the SE system is expected to shorten breeding cycles, facilitate propagation of superior cultivars, and support large-scale industrial applications in *C. intermedia*. Furthermore, the stable transformation system provides a platform for molecular breeding and gene function verification.

## 1. Introduction

In vitro plant culture techniques, including pollen embryogenesis, organogenesis, and somatic embryogenesis (SE), are powerful biotechnological tools for enhancing productivity and improving agronomic traits in crops, ornamental plants, and forage species. Among these approaches, SE demonstrates exceptional potential for rapid, large-scale seedling cultivation [1]. SE is indispensable for plant genetic transformation and molecular breeding programs. This is because somatic embryos predominantly originate from single somatic cells, resulting in minimal chimera formation and high regeneration efficiency [2,3]. After somatic cells are induced to develop into embryonic cells, a series of morphological and biochemical changes occur, resulting in bipolar structures at root and stem ends independent of vascular connections to maternal tissues [4,5]. Many previous studies have shown that the development of somatic embryos is affected by biological factors, such as genotype, developmental stage, and explant type, and physical factors, such as light intensity, photoperiod, and thermal condition [6,7]. In addition, plant growth regulators (PGRs) with auxin and cytokinin as the main components and organic additives, such as casein hydrolysate, glutamine, proline, or polyamines, have been proven to play key roles in SE [8,9].

*Caragana intermedia* (syn. *C. liouana*), a perennial deciduous shrub in the genus *Caragana* (Fabaceae), serves multiple ecological functions, such as windbreak, sand fixing, mud interception, water storage, and soil amelioration [10,11]. It predominantly relies on sexual reproduction through seeds, while its asexual propagation techniques remain undeveloped. Establishing an asexual propagation (especially SE) is expected to substantially enhance the large-scale application and dissemination of elite cultivars. The SE in the genus *Caragana* was first reported by Ci et al. [12], who utilized plumular axes and cotyledons of *C. korshinskii* as induction explants. Subsequently, Shen et al. [13] successfully induced somatic embryos from immature cotyledons of *C. fruticosa*; however, both the induction efficiency and embryo yield were extremely low. So far, no studies have yet reported SE in *C. intermedia*.

Conventional breeding methods are time consuming and inefficient, largely failing to achieve rapid genetic improvement in *C. intermedia*. However, genetic transformation provides a feasible pathway to accelerate its genetic enhancement and develop elite cultivars with desirable traits, such as spineless, high protein, or low lignin content, which are preferable when *C. intermedia* is used as forage. Genetic transformation based on SE systems is a well-established method for producing transgenic materials. This technology provides an efficient platform to investigate plant gene functions and create novel germplasms via gene editing.

To date, few studies have been conducted on genetic transformation technologies for the genus *Caragana*. The Chinese Academy of Agricultural Sciences pioneered genetic transformation research in *C. korshinskii*, with PCR and Southern blot analyses confirming a transformation efficiency of only 0.5% [14]. However, the following development of an *Agrobacterium tumefaciens*-mediated callus transformation method still yielded suboptimal efficiency at 0.42% [15]. Subsequently, researchers established a hairy root transformation system using *Agrobacterium rhizogenes* strain K599 (OD₆₀₀ = 0.9), achieving a transformation efficiency of 33.7% [16]. Notably, the approach merely generates transgenic root systems without achieving full-plant transformation. Recently, using the embryonic tips of germinating seeds of *C. korshinskii* as explants, researchers achieved a 78% adventitious bud induction rate through direct organogenesis [17]. Based on this achievement, researchers successfully established an *Agrobacterium*-mediated genetic transformation system. Nevertheless, the transformation efficiency was still not high enough. Further research is still needed to improve the transformation efficiency.

To further advance the research on asexual propagation techniques of *C. intermedia* and facilitate genetic transformation and breeding processes, we developed an SE system. Key factors affecting SE efficiency were investigated, including explant developmental stages, PGR combinations, inoculation modes, and other factors. On this basis, a genetic transformation system was preliminarily established. This system demonstrates significant potential for advancing genetic improvement and germplasm innovation in *C. intermedia*.

## 2. Results

### 2.1. Effects of Cotyledon Inoculation Modes on SE

To evaluate SE induction efficiency based on cotyledon orientation, the dorsal and ventral surfaces of *C. intermedia* cotyledons were histologically analyzed (Supplementary Figure S1). Dorsal cells exhibited irregularly globular morphology, with multiple vacuoles and loose arrangement. In contrast, ventral cells had fewer vacuoles, higher chloroplast density, and compact cellular organization. Ventral surface contacting with induction medium resulted in a 56.50% (±5.45%) cotyledon-stage embryo induction rate and yielded 11.11 (±1.21) cotyledon-stage embryos per explant (number of cotyledon-stage embryos/total number of explants), representing 1.53-fold (induction rate) and 1.31-fold (embryo number) increases compared to dorsal surface exposure (Supplementary Table S1). Thus, maximal SE efficiency was achieved by orienting cotyledons with ventral surfaces in contact with the medium.

### 2.2. Effects of Cotyledon Development Stages on SE

To systematically assess SE competency, cotyledons were harvested from pods at defined developmental stages (1–10 weeks after flowering, WAF; see Supplementary Figure S2). As quantified in Table 1, SE induction failed in 1–3 WAF explants due to incomplete cotyledon differentiation or excessive tissue juvenility. At 4 WAF, initial SE activity was observed with a low induction rate (5.66 ± 0.22%) and embryo yield (0.87 ± 0.09 embryos/explant). By 5 WAF, the induction rate of cotyledon-stage embryos surged to 37.67% (±0.46%), with an average of 7.51 (±0.30) embryos per explant. Maximal induction efficiency occurred at 6–7 WAF, with a rate exceeding 55% and 9–12 cotyledon-stage embryos per cotyledon. These cotyledon-stage embryos further developed into complete plantlets after germination, confirming 6–7 WAF as the optimal period for SE induction. Starting from 8 WAF, cotyledons underwent senescence, with only localized regions retaining embryogenic competence, leading to a decrease in SE induction rate and embryo yield. At 9 WAF, the induction rate plummeted to 1.48% (38.6-fold reduction vs. 7 WAF), with embryo yield declining to 0.35 (±0.03) per explant (33.7-fold reduction vs. 7 WAF). By 10 WAF, cotyledons completely lost embryogenic competency and exhibited progressive tissue browning.

### 2.3. Effects of PGRs on SE

To assess the effects of plant growth regulators (PGRs) on SE, cotyledons were harvested at 5, 6, and 7 WAF for induction experiments. The results demonstrated that low concentrations of NAA and 6-BA significantly enhanced SE efficiency, whereas the cotyledon-stage embryo induction rate declined progressively with increasing PGR concentrations (Table 2). PGR combinations exhibited stage-dependent efficacy in SE, with significant variation across cotyledon developmental stages. At 5 WAF, treatment 1 achieved the highest cotyledon-stage embryo induction rate (79.17 ± 0.31%), while treatment 4 yielded the maximum embryo count (15.23 ± 0.78 embryos per explant). At 6 WAF, treatment 2 maximized both induction rate (65.00 ± 1.46%) and embryo yield (10.20 ± 0.46 embryos/explant). At 7 WAF, treatment 8 generated peak values of a 61.90% (±1.72%) induction rate and 15.63 (±0.75) embryos per explant. Higher PGR concentrations are required to induce embryogenesis in older cotyledons, as observed. Integrated analysis identified 5 mg/L NAA and 3 mg/L 6-BA as the optimal combination, yielding the highest mean induction rate (58.40 ± 1.83%) and embryo count (12.13 ± 0.86 per explant) across all stages.

According to the previous findings in this study (Section 2.2), cotyledons at the 6–7 WAF developmental stages were optimal explants. Inoculation of these explants onto the culture medium containing 5 mg/L NAA and 3 mg/L 6-BA resulted in highest somatic embryo induction rate and cotyledon-stage embryo count.

### 2.4. Development Progress of Embryos

**Figure 1:**
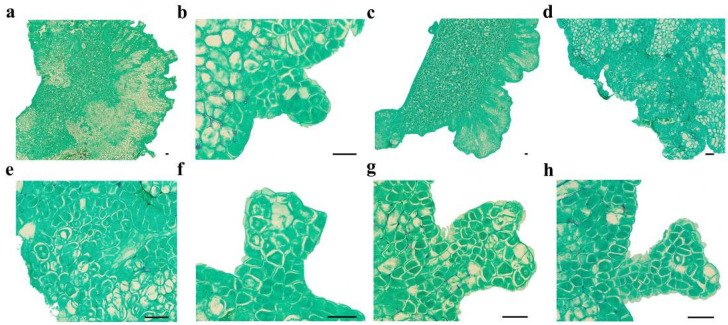
Microscopic observation of SE in *C. intermedia*. (**a**) Dorsal of cotyledon protrude outward and form globular embryos; (**b**) globular embryo; (**c**) calli formed on the dorsal edge of the cotyledon explant; (**d**) proembryonic cell mass; (**e**) amplification of proembryo; (**f**) heart-shaped embryo; (**g**) torpedo-shaped embryo; (**h**) cotyledon-stage embryo. Bar: 50 µm.

**Figure 2:**
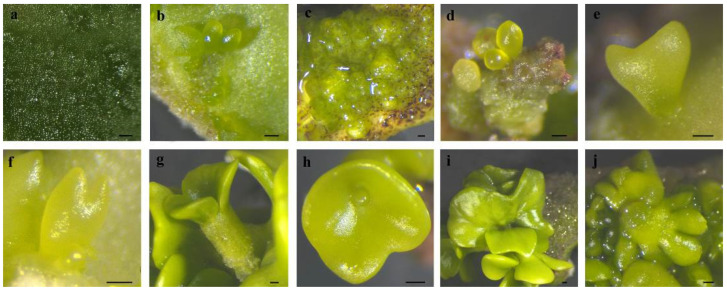
The SE process in *C. intermedia*. (**a**) The protuberances on cotyledon surface; (**b**) protuberances directly developed into globular embryos; (**c**) protuberances developed into calli; (**d**) calli differentiated into globular embryos; (**e**) heart-shaped embryo; (**f**) torpedo-shaped embryo; (**g**) mature cotyledon-stage embryo; (**h**) connate cotyledons; (**i**) asymmetrical cotyledons; (**j**) multiple cotyledons. Bar: 200 µm.

Cotyledons harvested at 6–7 WAF exhibited enlargement and protuberance (Figure 1a and Figure 2a) within 3 days of culture on an SE induction medium. SE proceeded via two distinct developmental pathways in *C. intermedia*. In Pathway I, globular embryos differentiated directly from cotyledon surfaces without callus intervention (Figure 1b and Figure 2b). In Pathway II, callus proliferation was initiated at cotyledon protuberances (Figure 1c and Figure 2c), serving as precursors for embryogenic structures. Proembryogenic cell aggregates emerged from callus tissues, characterized by densely cytoplasmic cells with prominent nuclei (Figure 1d,e). These proembryos underwent morphogenetic transition to globular-stage embryos (Figure 2d). Pathway I demonstrated accelerated morphogenesis, achieving globular embryo formation within 7 days post-inoculation (dpi). In Pathway II, callus initiation occurred at 6 dpi, with proembryogenic masses becoming evident by 8 dpi. Following 2–3 days of further morphogenesis, proembryos transitioned to globular-stage embryos. Subsequently, globular embryos proceeded through sequential developmental phases: heart-shaped (Figure 1f and Figure 2e), torpedo-shaped (Figure 1g and Figure 2f), and ultimately cotyledon-stage embryos (Figure 1h and Figure 2g–j). Some mature cotyledon-stage embryos were harvested at 15 dpi. At the cotyledonary stage, developmental abnormalities occurred concomitantly with normal embryogenesis; abnormal morphologies were observed alongside normal embryos, including connate cotyledons (Figure 2h), asymmetrical cotyledons (Figure 2i), and multiple cotyledons (≥3 cotyledons; Figure 2j).

### 2.5. Germination of Cotyledon-Stage Embryos

**Figure 3:**
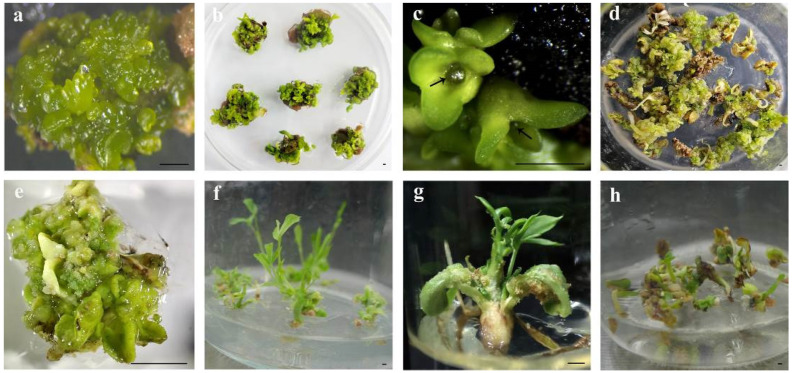
Germination of cotyledon-stage embryos on different media. (**a**) Immature cotyledon-stage embryos; (**b**) maturation culture of cotyledon-stage embryos; (**c**) mature cotyledon-stage embryos (arrows indicate the apical meristem); (**d**) somatic embryos develop into brown calli cultured for 20 days on the 1/2 MS medium supplemented with 0.005 mg/L NAA; (**e**) somatic embryos form calli following 15 days culture on the MS medium devoid of PGRs; (**f**,**g**) germinating somatic embryos after culture on the HS-MS medium for 20 days; (**h**) germinating somatic embryos after culture on the 1/4-strength MS medium for 20 days, exhibiting browning of embryo cotyledons. Bar: 1 mm.

Immature cotyledon-stage embryos (Figure 3a) required immediate transfer to the germination medium for maturation culture (Figure 3b). Apical meristems (black arrows in Figure 3c) in mature cotyledon-stage embryos would differentiate into plantlets under optimal culture conditions. The ½ MS medium supplemented with NAA (0.005, 0.01, and 0.05 mg/L) failed to induce germination, instead promoting callus formation and progressive tissue browning (Supplementary Table S2; Figure 3d). Both full-strength MS and 1/2 MS media (lacking PGRs) resulted in germination failure, with embryos developing into calli (Figure 3e). When all components in the MS medium were halved (Supplementary Table S3), the cotyledon-stage embryos were able to germinate normally and formed plantlets (Figure 3f,g) after about 20 days, with a germination rate of 34.36% (± 5.41%). The quarter-strength MS medium allowed limited germination (5.34 ± 0.98%), but surviving plantlets showed embryo cotyledon browning during subsequent subcultures (Figure 3h). Vigorously growing plantlets were transferred to the MS medium supplemented with 30 g/L sucrose and 7 g/L agar for further development into transplantable seedlings.

### 2.6. Genetic Transformation of C. intermedia

**Figure 4:**
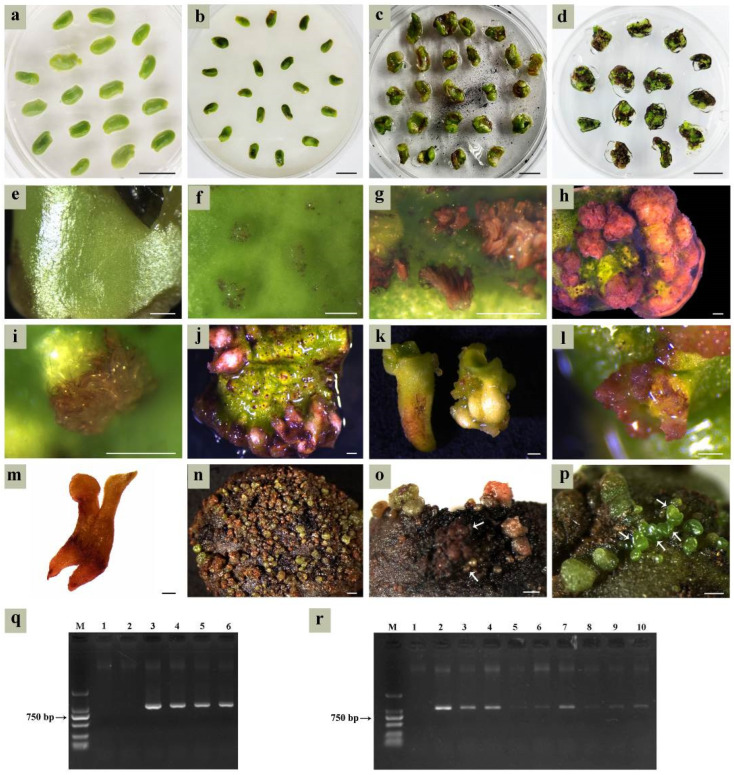
*Agrobacterium*-mediated genetic transformation based on the SE system. (**a**) Pre-culture of cotyledons on the SE induction medium; (**b**) co-culture of cotyledons with *Agrobacterium* on the SE induction medium; (**c**,**d**) somatic embryo screening after co-culture on the selection medium; (**e**) newly inoculated cotyledon; (**f**) transgenic protuberances; (**g**) transgenic tissue at 7 days post-infection (timing started from the end of co-culture); (**h**) transgenic embryogenic callus; (**i**) transgenic globular embryos at 10 days post-infection; (**j**,**k**) transgenic radicles; (**l**) transgenic cotyledon-stage embryos; (**m**) completely transgenic cotyledon-stage embryo; (**n**) massive transgenic globular embryos; (**o**) necrosis in transgenic embryos (indicated by the white arrows) and maternal explants during the globular embryo transformation stage; (**p**) untransformed somatic embryos; (**q**) PCR analysis of transgenic calli, M was a DL2000 marker, lanes 1 and 2 were the negative control (untransformed calli), lane 3 was the positive control (*pDR5:RUBY* plasmid), lanes 4–6 were red transgenic calli; (**r**) PCR analysis of transgenic cotyledon-stage embryos, M was a DL2000 marker, lane 1 was untransformed cotyledon-stage embryos, lanes 2–10 were the red transgenic cotyledon-stage embryos. Bar: 1 cm (**a**–**d**), 500 µm (**e**–**p**).

Cotyledons harvested at 6–7 WAF were inoculated on the SE induction medium and pre-cultured for 3 days (Figure 4a). Following *Agrobacterium* infection, explants underwent co-culture for 3 days (Figure 4b) before transferring to the selection medium. With prolonged screening time, parts of the cotyledons’ tissue gradually turned brown (Figure 4c), and only a small portion developed into cotyledon-stage embryos (Figure 4d). Newly inoculated cotyledons displayed smooth epidermal surfaces (Figure 4e), while following co-culture, a large number of protuberances on the cotyledons’ surface showed up after being transformed by *A. tumefaciens* (Figure 4f). The transgenic tissues were clearly visible as red pigmentation for approximately 7 days post-infection (Figure 4g). By 10 days post-infection, transgenic protuberances differentiated into either callus clusters (Figure 4h) or globular embryos (Figure 4i). Only the top cells of globular embryos were transformed; however, later-stage differentiated cells failed to be transformed.

Due to the uncertain developmental fate of the embryogenic callus, the transgenic regions of embryos might be radicles (Figure 4j,k), cotyledons (Figure 4l), or entire embryos (Figure 4m). The probability of the whole embryo being transformed was low, with most exhibiting only localized infection. Despite the transformation rate exceeding 50% at the globular embryo stage (Figure 4n), most transgenic globular embryos arrested development. Extended culture periods triggered synchronized necrosis in both transgenic embryos and maternal explants (Figure 4o). Non-necrotic cotyledons retained embryogenic competence to regenerate new embryos (Figure 4p), which complicated the identification of transgenic materials that could not be visually identified.

### 2.7. Identification of Transgenic Materials

Three transgenic red calli, two untransformed calli, and a *pDR5:RUBY* plasmid were subjected to PCR analysis. The same amplicons (1008 bp) were detected in a *pDR5:RUBY* plasmid and all red calli, while untransformed calli showed no amplification (Figure 4q). Next, nine transgenic red and one untransformed cotyledon-stage embryo were subjected to PCR analysis. All red embryos exhibited *RUBY*-specific amplicons with variable band intensities (Figure 4r). Collectively, the results from phenotypic observation and PCR analysis confirmed successful *RUBY* transformation in somatic embryos. Unfortunately, we failed to obtain plantlets from transgenic cotyledon-stage embryos during subsequent culturing.

### 2.8. TRV-VIGS System Verification

**Figure 5:**
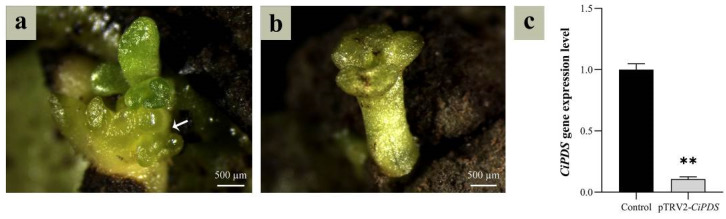
Identification of *CiPDS* interfering embryos. (**a**) *CiPDS*-silenced somatic embryos (indicated by the white arrow); (**b**) *CiPDS*-silenced cotyledon-stage embryos; (**c**) expression level of *CiPDS*-silenced cotyledon-stage embryo, and the control was a cotyledon-stage embryo transformed with a pTRV2 empty vector. The asterisks (**) indicate a significant difference at the 0.01 significance level according to Student’s *t*-test.

To validate the transformation protocol, cotyledons were agroinfiltrated with *Agrobacterium* suspensions carrying pTRV1 + pTRV2 (empty vector control) or pTRV1 + pTRV2-*CiPDS* (target gene silencing construct) using the TRV-VIGS system. Photobleached somatic embryos (yellow phenotype, Figure 5a) emerged and differentiated into cotyledon-stage embryos (Figure 5b). RT-qPCR analysis demonstrated a 9.35-fold downregulation of *CiPDS* expression in yellow embryos versus control (*p* < 0.01, Student’s *t*-test; Figure 5c), conclusively validating the efficacy of this SE-based transformation system in *C. intermedia*.

## 3. Discussion

As a critical component of seed embryos, cotyledons serve dual roles in nutrient storage (e.g., lipids, proteins) and the protection of embryonic structures (plumule, radicle, and hypocotyl). Immature cotyledons are established explants for somatic embryogenesis (SE), as demonstrated across diverse plant species [18,19,20]. SE is the developmental reprogramming of somatic cells into embryos in higher plants, a complex molecular and biochemical process based on cell totipotency [21]. Under identical genotypes, explant type, and culture conditions, somatic embryo induction depends largely on the specific developmental stages of tissues [22,23]. The induction rate and embryo yields differed significantly among the cotyledons of *C. intermedia* collected at different developmental stages under the same culture conditions. Cotyledons that are juvenile (<4 WAF) or senescent (>8 WAF) result in reduced somatic embryo induction or even no somatic embryos. Senescent cotyledons showed limited embryogenic competence due to diminished cellular plasticity and prolonged reprogramming timelines [21]. In contrast, immature cotyledons (5–7 WAF) exhibit enhanced embryogenic potential because they are in a period of vigorous development with a high capacity for cell division and regeneration.

SE proceeds via two distinct pathways: direct somatic embryogenesis (DSE) from explants or indirect somatic embryogenesis (ISE) through callus intermediates [24]. DSE bypasses callus formation, generating embryos directly from epidermal/subepidermal cell masses of explants [25]. In contrast, ISE requires callus induction to establish embryogenic cells prior to embryo differentiation, demanding stringent control of auxin/cytokinin ratios [26]. The embryogenic ability of callus in the ISE pathway is different. Only the globular callus with a soft surface (containing embryogenic cells) can acquire embryogenic ability, while the rough, dry, and brittle callus cannot [27,28]. The embryogenic cells are characterized by a thick cell wall, denser cytoplasm, fragmented vacuoles, a large nucleolus, a highly active nucleus, a high nucleus-to-cytoplasm ratio, and a low level of heterochromatin [29,30]. Histological analysis confirmed the co-occurrence of DSE and ISE pathways in *C. intermedia* somatic embryos. The time required for the formation of a cotyledon-stage embryo through the DSE pathway was 15 days, and that through the ISE pathway was at least 18 days. In the process of SE in plants, cotyledon-stage embryos are formed in two ways, which can improve the utilization rate of explants and increase the yield of the cotyledon-stage embryos, thus facilitating the propagation and production of plantlets.

Somatic embryos develop a bipolar embryonic axis containing shoot apical meristem (SAM) and root apical meristem (RAM) during SE progression [23]. These embryos establish an autonomous vascular system lacking connectivity to maternal explants. Both SAM and RAM maintain independent meristematic activity throughout early developmental stages [31]. In *C. intermedia*, SE originated primarily from cotyledon epidermal cells, proceeding through the typical globular, heart, torpedo, and early cotyledonary embryo stages. Based on morphological and histological analyses during SE, we speculate that embryos may remain tightly connected to the cotyledon explants prior to maturation, with no functional vascular system observed at this stage. However, when transformed into a mature cotyledon-stage embryo, an abscission layer differentiates between the embryo and explant, retaining only the basal vascular tissue to mediate nutrient and water transport.

Not all embryogenically competent embryos develop into plantlets [32]. Cotyledon-stage embryos require a maturation phase to acquire germination competence, which is further modulated by embryo morphology and culture parameters. Morphologically normal cotyledon-stage embryos exhibit bilateral symmetry with two expanded cotyledons and a distinct radicle. Numerous researchers have found that SE systems frequently generate abnormal embryos, including elongated hypocotyls, cup-shaped or fused cotyledons, absent leaf primordia, and apical meristem deformation [33,34]. Such morphological anomalies significantly impair germination efficiency and compromise conversion to viable plantlets. In *C. intermedia*, abnormal cotyledon-stage embryos, such as connate cotyledons, asymmetrical cotyledons, and multiple cotyledons, also existed during SE, which affected the acquisition of subsequent plantlets.

*Agrobacterium*-mediated genetic transformation has become the most commonly used method for plant genetic transformation due to its advantages of high transformation efficiency, low cost, and precise integration of defined T-DNA [35]. Under an efficient regeneration system, plant genetic transformation can be affected by *Agrobacterium* strain, bacterial density, infection duration, antibiotic type and concentration, bacteriostatic agent type and concentration, and operation procedure [36,37]. Among these, the appropriate *Agrobacterium* strain and infection solution concentration are important factors to promote the success of transformation [35,38]. Co-culture is a critical step in plant genetic transformation, during which T-DNA is inserted into the plant genome [39]. Prolonged co-cultivation induces *A. tumefaciens* overgrowth, which is harmful to plant cells [40,41]. The use of antibiotics can reduce false positives in transformed materials and screening workload while improving genetic transformation efficiency [38]. However, antibiotics can also cause damage to plant tissue, thereby reducing transformation efficiency.

Taking these factors into consideration, we have preliminarily established the genetic transformation system. *A. tumefaciens* suspensions (OD600 = 0.5) were used to infect the explants. After 3 days of co-cultivation under dark conditions, somatic embryo screening was conducted. Cefotaxime (400 mg/L) was added as a bacteriostatic agent, and hygromycin (13 mg/L) was used as a selection antibiotic. The results indicated that this combination suppressed *A. tumefaciens* growth for 15 days. However, residual *A. tumefaciens* exhibited regrowth beyond day 15, causing bacterial biofilm formation and progressive necrosis of transgenic tissues. Additionally, both the bacteriostatic agent and antibiotic reduced somatic embryo differentiation frequency, caused explant oxidative browning, and declined embryogenesis efficiency. Thus, the primary focus of future research on genetic transformation in *C. intermedia* should be optimizing *Agrobacterium* control through the rational adjustment of infection solution concentration, co-culture duration, and bacteriostatic agent dosage. Following *Agrobacterium* inhibition, the subsequent step involves screening antibiotic types and concentrations.

## 4. Materials and Methods

### 4.1. Seed Developmental Progression

*C. intermedia* plants cultivated in Horinger County, Hohhot City, Inner Mongolia Autonomous Region, China (40°30′24.49″ N, 111°50′10.60″ E, altitude 1161.3 m) were used as experimental material. In order to identify optimal explants (immature cotyledons) for SE based on their appearance, the developmental progression of seeds within *C. intermedia* pods was systematically monitored. At each developmental phase, immature seeds were photographed and status recorded, and the length and width of fifty randomly selected specimens were measured.

### 4.2. Somatic Embryo Induction

Plump and insect-free pods (1–10 weeks after flowering, WAF) were collected. Intact seeds were picked out, rinsed under running tap water for 20 min, surface-sterilized with 70% (*v*/*v*) ethanol for 30 s in a laminar flow cabinet, followed by 2% (*v*/*v*) sodium hypochlorite (NaClO) for 4 min, and finally rinsed four times with sterile distilled water. Sterilized seeds were aseptically transferred to sterile filter paper, and a longitudinal incision was made along the dorsal surface using a sterile scalpel to isolate embryos. Isolated embryos were dissected to remove the plumule, hypocotyl, and radicle, retaining only cotyledons for culture initiation. Cultures were maintained at 25 ± 1 °C under a 16/8 h light/dark photoperiod with a photosynthetic photon flux density (PPFD) of 100 μmol·m^−2^·s^−1^. To evaluate the effect of explant orientation on embryogenesis, cotyledons were positioned with either the dorsal or ventral surface in contact with the culture medium. The culture media were replaced weekly to prevent nutrient depletion. Each experimental treatment comprised three biological replicates, with 30 cotyledons per replicate. Components of the culture media for specific experimental objectives are detailed in Table 3, with the basal medium formulation provided in Supplementary Table S3. Following 20 days of induction, the number of cotyledon-stage embryos (number of cotyledon-stage embryos/total number of explants) was quantified, and the cotyledon-stage embryo induction rate was calculated.

Cotyledon-stage embryo induction rate (%) = number of explants producing cotyledon-stage embryo/total number of inoculated cotyledons × 100%.

### 4.3. Paraffin Section

Tissues were fixed over 24 h in an FAA solution (formaldehyde–acetic acid–alcohol; 70% ethanol:glacial acetic acid:38% formaldehyde = 90:5:5, *v*/*v*/*v*, supplemented with 5% (*v*/*v*) glycerol). Then, samples were dehydrated through a graded ethanol series (70%, 85%, 95%, and 100%, *v*/*v*). Dehydrated tissues were cleared in xylene, infiltrated with molten paraffin, and embedded in paraffin wax. After paraffin solidification, blocks were sectioned into 8 μm thick slices using a Leica RM2235 rotary microtome (Leica Biosystems, Wetzlar, Germany). Sections were mounted onto glass slides and placed at 56 °C to stretch. Following slide drying and dewaxing with xylene, sections were stained with Safranin-Fast Green and permanently mounted with a resinous mounting medium for microscopic observation.

### 4.4. Genetic Transformation

#### 4.4.1. Reporter Genes

In this study, the *RUBY* and *PDS* (*phytoene desaturase*) were selected as reporter genes. Transgenic plants expressing *RUBY* accumulate betalains, resulting in visible red pigmentation of plant tissues [42]. The *PDS*-encoded protein is localized to the chloroplast thylakoid membrane and plays a photoprotective role in chloroplast function [43]. TRV-VIGS (tobacco rattle virus-based virus-induced gene silencing)-mediated silencing of *PDS* disrupts carotenoid biosynthesis and metabolism, leading to chlorophyll degradation and resulting in photobleaching and chlorosis symptoms due to impaired photoprotection [44]. The distinct visual phenotypes conferred by these reporter genes (*RUBY*-induced red pigmentation and *PDS*-silencing-induced chlorosis) enable rapid, non-destructive assessment of transformation success. Therefore, they were selected as reporter genes for screening successful genetic transformation events.

#### 4.4.2. Activation of Agrobacterium

The *A. tumefaciens* GV3101 strains harboring *pDR5:RUBY*, pTRV1, pTRV2, or pTRV2-*CiPDS* plasmid were individually streaked onto LB agar plates (medium composition detailed in Supplementary Table S4) supplemented with 50 µg/mL kanamycin (Kan) and 25 µg/mL gentamicin (Gm), followed by incubation at 28 °C for 36–48 h. Five single colonies were selected and inoculated into 2 mL of an LB liquid medium containing identical antibiotic concentrations (50 µg/mL Kan, 25 µg/mL Gm) and 50 µM acetosyringone. Primary culture was incubated at 28 °C with shaking at 250 rpm for 24 h until the bacterial culture reached saturation. A total of 1 mL inoculum from the primary culture was transferred to 100 mL of a fresh LB medium supplemented with antibiotics (50 µg/mL Kan, 25 µg/mL Gm) and 20 µM AS. Secondary culture was incubated at 28 °C with shaking at 250 rpm until the optical density at 600 nm (OD600) reached 0.5–0.8 (about 8 h).

#### 4.4.3. Preparation of Infective Bacterial Suspension

Thirty-five milliliters of the bacterial culture were transferred to a fifty milliliter sterile centrifuge tube and centrifuged at 4000 rpm for 20 min at 6 °C. Within a laminar flow cabinet, the supernatant was discarded, and pellets were resuspended in sterilized resuspension solution (MS basal medium + 5 mg/L NAA + 3 mg/L 6-BA + 30 g/L sucrose + 200 µM AS (filter-sterilized) + 0.001% Silwet-77 (filter-sterilized), pH 5.4) was added. The bacterial pellet was thoroughly resuspended by vortex mixing, and the OD_600_ was adjusted to 0.5 with a sterilized resuspension solution. For TRV-VIGS-mediated functional validation, *A. tumefaciens* suspensions harboring pTRV1 and pTRV2-*CiPDS* were mixed at a 1:1 (*v*/*v*) ratio prior to plant inoculation. A 1:1 mixture of pTRV1 and pTRV2 (empty vector) served as the control treatment.

#### 4.4.4. Cotyledons Agroinfection

**Figure 6:**
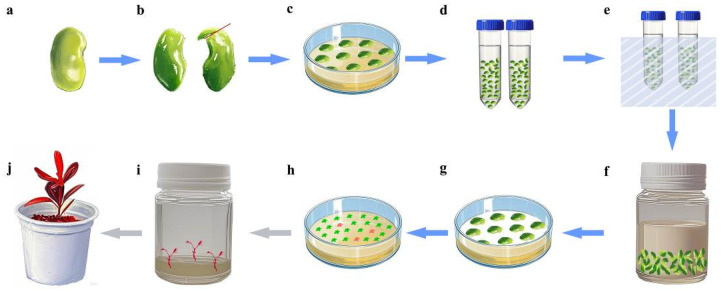
*Agrobacterium*-mediated SE transformation workflow. (**a**) Seed after 6–7 weeks of flowering; (**b**) cotyledon explant (red line shows the incision location); (**c**) pre-culture; (**d**) treatment with mannitol; (**e**) sonication; (**f**) *Agrobacterium tumefaciens* infection; (**g**) co-culture; (**h**) somatic embryo induction and screening (red blocks indicate transgenic embryos); (**i**) germination of transgenic embryos; (**j**) transgenic seedling.

A schematic workflow of *Agrobacterium*-mediated SE transformation is provided in Figure 6. The cotyledons of the optimal developmental stage were pre-cultured on the optimal somatic embryo induction medium for 3 days. Then, the cotyledons were pretreated in a sterilized 0.4 M mannitol solution and incubated at 25 °C under gentle shaking (160 rpm) for 24 h. Under aseptic conditions, cotyledons were soaked in the fresh resuspension solution. Brief sonication (30 s at 100 kHz) was applied to enhance bacterial adherence, followed by removal of the suspension. Then, *Agrobacterium* infection suspension (OD₆₀₀ = 0.5) was applied to completely submerge the explants. Co-cultivation was performed under gentle shaking (100 rpm) at 25 °C for 30 min.

#### 4.4.5. Co-Culture and Somatic Embryos Screening

Following co-cultivation, residual *Agrobacterium* suspension was blotted from cotyledon surfaces using sterile filter paper. Cotyledons were positioned ventral surface down on sterile filter paper overlaying the co-culture medium (Table 3, 4^#^). Co-cultured cotyledons were maintained in darkness at 25 °C for 3 days. Post-co-culture, explants were immersed in 2500 mg/L cefotaxime for 30 min to eliminate residual bacteria, followed by four sequential rinses with sterile distilled water. Finally, explants were transferred to the somatic embryo selection medium (Table 3, 5^#^) and cultured under a 16 h photoperiod (100 μmol·m^−2^·s^−1^). The culture medium was changed weekly until cotyledon-stage somatic embryos developed. To suppress phenolic browning, 1 mg/L activated charcoal was incorporated into the fresh medium during subculturing. Upon reaching the mature cotyledonary stage, somatic embryos were transferred to the differentiation medium (Table 3, 6^#^) to promote the development of radicle, hypocotyl, and plumule tissues.

### 4.5. Identification of Transgenic Material

#### 4.5.1. RNA Extraction, cDNA Synthesis, and PCR Analysis

Primary screening was performed via phenotypic observation of *RUBY*-induced red pigmentation or *PDS*-silencing-induced chlorosis. Putative transgenic lines from the primary screen were subjected to RNA extraction.

Total RNA was isolated from frozen plant tissue using the Quick-RNA™ Plant Miniprep Kit (Zymo Research Corporation, Orange County, CA, USA). On-column DNase I treatment was performed prior to RNA clean-up to eliminate genomic DNA contamination. RNA concentration and purity were determined using an ultra-micro spectrophotometer (Quawell Q5000, Quawell Technology Inc., San Jose, CA, USA), with A260/A280 and A260/A230 ratios both between 1.8 and 2.2, indicating high purity. RNA integrity was further verified by 1.0% agarose gel electrophoresis. High-quality RNA samples were treated with gDNA Eraser™ (Takara Bio Technology (Beijing) Co., Ltd., Beijing, China) at 42 °C for 5 min to remove residual genomic DNA, followed by cDNA synthesis using the PrimeScript™ II 1st Strand cDNA Synthesis Kit (Takara Bio Technology (Beijing) Co., Ltd., Beijing, China) according to the manufacturer’s protocol. The synthesized cDNA was stored at −80 °C. Gene-specific primers (Supplementary Table S5) used in PCR analysis were designed using Primer Premier 5.0 (Premier Biosoft, Palo Alto, CA, USA). PCR products were resolved by electrophoresis on a 1% agarose gel.

#### 4.5.2. Real-Time Quantitative Polymerase Chain Reaction

The synthesized cDNA was diluted 16-fold (1:16) to serve as a template for RT-qPCR analysis. RT-qPCR reactions were conducted using TB Green^®^ Premix Ex Taq™ II (Tli RNaseH Plus) (Takara Bio Technology (Beijing) Co., Ltd., Beijing, China) on a qTOWER 3G real-time PCR system (Analytik Jena GmbH, Jena, Thuringia, Germany). Each RT-qPCR reaction mixture (20 μL total volume) contained 10 μL TB Green Premix Ex Taq II (Tli RNaseH Plus), 0.8 μL each of 10 μM forward and reverse primers (Primer F/R, Supplementary Table S5), 5 μL 16-fold diluted cDNA template, and 3.4 μL RNase-free ddH₂O. The thermal cycling conditions were initial pre-denaturation at 95 °C for 5 min, 40 cycles of denaturation at 95 °C for 10 s, annealing at 58 °C for 20 s, and extension at 72 °C for 15 s, followed by a dissociation curve analysis stage (95 °C for 15 s, 65 °C for 1 min, 97 °C for 1 s, 40 °C for 10 s, and 60 °C for 15 s). All reactions included triplicate technical replicates. The relative transcript levels of target genes were calculated using the 2^−ΔΔCT^ method [45], with *CiEF1α* as the endogenous reference gene for RT-qPCR data analysis.

### 4.6. Image Collection and Statistics

Seed and somatic embryo images were captured using a SteREO Discovery.V8 stereomicroscope (Carl Zeiss AG, Oberkochen, Baden-Württemberg, Germany) coupled with an AxioCam MRc5 digital camera system. Paraffin sections were imaged with an Olympus BX41 compound microscope equipped with a DP74 digital camera (Olympus Corp., Tokyo, Japan). Statistical analyses were conducted with IBM SPSS Statistics 20 (IBM Corp., Armonk, NY, USA). One-way ANOVA followed by Duncan’s multiple range test (α = 0.05) was employed for comparisons among three or more experimental groups. Student’s *t*-test (two-tailed, α = 0.05) was utilized to assess significant differences between two experimental groups. Microsoft Excel 2010 (Microsoft Corp., Redmond, WA, USA) was used for data organization and graph generation. Figures were assembled using Adobe Photoshop CS3 (Adobe Systems Inc., San Jose, CA, USA).

## 5. Conclusions

In summary, we employed immature cotyledons as explants to investigate the effects of inoculation modes, cotyledon developmental stages, and PGR concentrations on embryo induction. Following the establishment of a somatic embryogenesis system, a genetic transformation protocol was preliminarily explored, resulting in the production of transgenic calli and cotyledon-stage embryos. This study provides a reference for research on propagation, genetic improvement, and new variety breeding in *C. intermedia*.

## Figures and Tables

**Table 1:** Effects of seed development stages on cotyledon-stage embryo induction.

Weeks After Flowering (WAF)	Seed Length (mm)	Seed Width (mm)	Cotyledon-Stage Embryo Induction Rate (%)	Number of Cotyledon-Stage Embryos
1	1.52 ± 0.05 h	0.72 ± 0.01 i	0.00 g	0.00 e
2	1.78 ± 0.02 g	1.09 ± 0.03 h	0.00 g	0.00 e
3	4.98 ± 0.04 f	1.99 ± 0.06 g	0.00 g	0.00 e
4	5.57 ± 0.11 e	2.85 ± 0.05 f	5.66 ± 0.22 e	0.87 ± 0.09 d
5	7.05 ± 0.11 d	4.16 ± 0.03 d	37.67 ± 0.46 c	7.51 ± 0.30 c
6	7.55 ± 0.01 c	4.33 ± 0.02 c	55.00 ± 0.48 b	9.58 ± 0.09 b
7	8.37 ± 0.02 b	4.58 ± 0.05 b	57.14 ± 0.40 a	11.80 ± 0.42 a
8	8.89 ± 0.05 a	4.86 ± 0.07 a	12.31 ± 0.17 d	1.25 ± 0.05 d
9	9.02 ± 0.05 a	4.96 ± 0.01 a	1.48 ± 0.18 f	0.35 ± 0.03 e
10	7.02 ± 0.13 d	3.41 ± 0.05 e	0.00 g	0.00 e

Note: Data are presented as means ± standard deviation. Statistical analysis was performed using one-way ANOVA, followed by Duncan’s multiple range test. Different lowercase letters in the same column denote significant differences (*p* < 0.05).

**Table 2:** Effectiveness of different PGR combinations on SE.

Treatment Number	NAA (mg/L)	6-BA (mg/L)	Cotyledon-Stage Embryo Induction Rate (%)				Number of Cotyledon-Stage Embryos			
			5 WAF	6 WAF	7 WAF	Means	5 WAF	6 WAF	7 WAF	Means
1	3	3	79.17 ± 0.31 a	53.89 ± 0.79 b	34.72 ± 1.30 e	55.93 ± 6.45 b	11.98 ± 0.95 bc	8.38 ± 0.23 bc	11.56 ± 0.39 bc	10.64 ± 0.64 b
2	3	5	58.33 ± 1.08 c	65.00 ± 1.46 a	41.67 ± 1.03 cd	55.00 ± 3.52 bc	10.04 ± 0.10 d	10.20 ± 0.46 a	6.88 ± 0.97 e	9.04 ± 0.62 cd
3	3	7	54.17 ± 0.60 cd	40.74 ± 1.60 c	30.56 ± 1.24 e	41.82 ± 3.47 f	10.61 ± 0.47 cd	7.55 ± 0.65 c	5.35 ± 0.44 e	7.84 ± 0.81 ef
4	5	3	64.88 ± 1.43 b	55.56 ± 1.78 b	54.76 ± 1.85 b	58.40 ± 1.83 a	15.23 ± 0.78 a	9.82 ± 0.55 ab	11.35 ± 0.48 bc	12.13 ± 0.86 a
5	5	5	37.67 ± 2.50 e	55.00 ± 0.90 b	57.14 ± 0.73 b	49.94 ± 3.18 e	7.51 ± 0.24 e	9.58 ± 0.54 ab	11.80 ± 0.49 bc	9.63 ± 0.66 c
6	5	7	51.33 ± 0.72 d	25.56 ± 1.93 d	40.21 ± 0.44 d	39.03 ± 3.78 g	8.33 ± 0.07 e	5.89 ± 0.55 de	10.30 ± 0.39 cd	8.17 ± 0.67 de
7	7	3	54.53 ± 1.23 cd	43.06 ± 1.05 c	55.83 ± 2.56 b	51.14 ± 2.21 de	12.74 ± 0.94 b	6.85 ± 0.75 cd	12.34 ± 0.92 b	10.64 ± 1.05 b
8	7	5	67.50 ± 1.80 b	28.61 ± 0.96 d	61.90 ± 1.72 a	52.67 ± 6.12 cd	14.83 ± 0.41 a	5.05 ± 0.08 e	15.63 ± 0.75 a	11.84 ± 1.72 a
9	7	7	40.00 ± 1.75 e	25.28 ± 0.26 d	46.16 ± 2.13 c	37.15 ± 3.20 g	4.87 ± 0.20 f	7.01 ± 0.20 cd	9.28 ± 0.23 d	7.05 ± 0.65 f

Note: Data are presented as means ± standard deviation. Statistical analysis was performed using one-way ANOVA, followed by Duncan’s multiple range test. Different lowercase letters in the same column denote significant differences (*p* < 0.05).

**Table 3:** Culture medium components under different research purposes.

Number	Research Purpose	Medium Components
1^#^	Effects of cotyledon inoculation modes on SE	MS + 5 mg/L NAA + 5 mg/L 6-BA + 30 g/L sucrose + 7 g/L agar + 500 mg/L hydrolyzed casein, pH 5.8
2^#^	Effects of different PGR combinations on SE	MS + (3, 5, 7) mg/L NAA + (3, 5, 7) mg/L 6-BA + 30 g/L sucrose + 7 g/L agar + 500 mg/L hydrolyzed casein, pH 5.8
3^#^	Germination of cotyledon-stage embryos	1/2 MS + (0, 0.005, 0.05, 0.01) mg/L NAA + 30 g/L sucrose + 7 g/L agar, pH 5.8;
		MS, HS-MS, 1/4 MS + 30 g/L sucrose + 7 g/L agar, pH 5.8
4^#^	Co-culture	MS + 5 mg/L NAA + 3 mg/L 6-BA + 8 g/L agar + 30 g/L sucrose + 200 µM AS, pH 5.4
5^#^	Somatic embryo selection	MS + 5 mg/L NAA + 3 mg/L 6-BA + 8 g/L agar + 30 g/L sucrose + 500 mg/L hydrolyzed casein + 400 mg/L Cef + 13 mg/L Hyg, pH 5.8
6^#^	Cotyledon-stage embryo differentiation	HS-MS + 30 g/L sucrose + 7 g/L agar + 250 mg/L Cef + 50 mg/L Kan, pH 5.8

Notes: HS-MS (half-strength Murashige and Skoog) medium: MS medium with all nutrients diluted to half concentration. 1/4 MS (1/4-strength MS) medium: MS medium with all nutrients reduced to one-quarter concentration. PGR: plant growth regulator, NAA: α-naphthaleneacetic acid, 6-BA: 6-benzylaminopurine, Cef: cefotaxime, Hyg: hygromycin, Kan: kanamycin, AS: acetosyringone. The ‘#’ symbol is used here as a culture medium identifier.

## Data Availability

The authors confirm that the data supporting the findings of this study are available within the article and its Supplementary Materials.

## Supplementary Materials

The following supporting information can be downloaded at https://www.mdpi.com/article/10.3390/plants14101545/s1, Figure S1: Cotyledon section; Figure S2: Morphological changes in *C. intermedia* seeds during development; Table S1: Effects of the modes of cotyledon contacting medium on cotyledon-stage embryo induction; Table S2: Germination of cotyledon-stage embryos; Table S3: Basic medium components; Table S4: LB basic medium components; Table S5: Primer sequences used in this study.

## References

[1] Ren Y., Yu X., Xing H., Tretyakova I.N., Nosov A.M., Yang L., Shen H. (2022). Interaction of subculture cycle, hormone ratio, and carbon source regulates embryonic differentiation of somatic cells in *Pinus koraiensis*. Forests.

[2] Tomiczak K., Mikuła A., Niedziela A., Wójcik-Lewandowska A., Domżalska L., Rybczyński J.J. (2019). Somatic embryogenesis in the family gentianaceae and its biotechnological application. Front. Plant Sci..

[3] Kamle M., Baek K.H. (2017). Somatic embryogenesis in guava (*Psidium guajava* L.): Current status and future perspectives. 3 Biotech.

[4] Quiroz-Figueroa F.R., Rojas-Herrera R., Galaz-Avalos R.M., Loyola-Vargas V.M. (2006). Embryo production through somatic embryogenesis can be used to study cell differentiation in plants. Plant Cell Tissue Organ Cult..

[5] Elhiti M., Stasolla C. (2022). Transduction of signals during somatic embryogenesis. Plants.

[6] Kaviraj C.P., Kiran G., Venugopal R.B., Kishor P.K., Rao S. (2006). Somatic embryogenesis and plant regeneration from cotyledonary explants of green gram [*Vigna radiata* (L.) Wilczek.]—A recalcitrant grain legume. Vitr. Cell. Dev. Biol..

[7] Sivakumar P., Gnanam R., Ramakrishnan K., Manickam A. (2010). Somatic embryogenesis and regeneration of *Vigna radiata*. Biol. Plant..

[8] de Melo Souza J.M., de Oliveira C.R., da Rocha Tavano E.C., Soriano L., Martinelli A.P., Ramírez-Mosqueda M.A. (2022). Somatic embryogenesis in citrus (*Citrus* spp.), var. Valencia. Somatic Embryogenesis.

[9] Sivanesan I., Nayeem S., Venkidasamy B., Kuppuraj S.P., RN C., Samynathan R. (2022). Genetic and epigenetic modes of the regulation of somatic embryogenesis: A review. Biol. Futur..

[10] Liu K., Yang Q., Yang T., Yang F., Wang R., Cong J., Li G. (2021). Transcriptome-based identification and expression profiling of AP2/ ERF members in *Caragana intermedia* and functional analysis of CiDREB3. Mol. Biol. Rep..

[11] Wan Y., Mao M., Wan D., Yang Q., Yang F., Mandlaa, Li G., Wang R. (2018). Identification of the *WRKY* gene family and functional analysis of two genes in *Caragana intermedia*. BMC Plant Biol..

[12] Ci Z., He X., He L., Huang K. (1998). The callus culture and redifferentiation of *Caragana korshinkii* Kom. J. Inner Mong. For. Coll..

[13] Shen H., Zhai X., Yang L. (2011). Somatic embryogenesis and plant regeneration of *Caragana fruticosa* from cotyledons of its young zygotic embryos. Plant Physiol. J..

[14] Qiu F., Gao H.W., Xu L. RNAi vector construction, *Agrobacterium* transformation and transgenic plant detection based on *PAL* gene of *Caragana korshinkii*. Proceedings of the 8th Symposium of Agricultural Biochemistry and Molecular Biology Branch of Chinese Society of Biochemistry and Molecular Biology.

[15] Hu J. (2014). Establishment of the Transformation System of *Caragana korshinskii* Kom. and Identification of *CkF5H* Transgenic *Arabidopsis thaliana*. Master’s Thesis.

[16] Yang C. (2021). Establishment of Genetic Transformation System of Hairy Roots of *Caragana intermedia* and Its Optimization. Master’s Thesis.

[17] Liu B., Shang X., Zhang X., Shao W., Ren L., Li G., Zhu M., Wang R. (2023). In vitro regeneration and *Agrobacterium*-mediated genetic transformation of *Caragana korshinskii*. For. Res..

[18] Hiraga S., Minakawa H., Takahashi K., Takahashi R., Hajika M., Harada K., Ohtsubo N. (2007). Evaluation of somatic embryogenesis from immature cotyledons of Japanese soybean cultivars. Plant Biotechnol..

[19] Rathore J.S., Rai M.K., Shekhawat N.S. (2012). Induction of somatic embryogenesis in gum arabic tree [*Acacia senegal* (L.) Willd.]. Physiol. Mol. Biol. Plants.

[20] Raza G., Singh M.B., Bhalla P.L. (2019). Somatic embryogenesis and plant regeneration from commercial soybean cultivars. Plants.

[21] Karami O., Aghavaisi B., Mahmoudi Pour A. (2009). Molecular aspects of somatic-to-embryogenic transition in plants. J. Chem. Biol..

[22] von Arnold S., Sabala I., Bozhkov P., Dyachok J., Filonova L. (2002). Developmental pathways of somatic embryogenesis. Plant Cell Tissue Organ Cult..

[23] Rai M.K., Akhtar N., Jaiswal V.S. (2007). Somatic embryogenesis and plant regeneration in *Psidium guajava* L. cv. Banarasi local. Sci. Hortic..

[24] 27 Yang X., Zhang X. (2010). Regulation of somatic embryogenesis in higher plants. Crit. Rev. Plant Sci..

[25] Pulianmackal A.J., Kareem A.V., Durgaprasad K., Trivedi Z.B., Prasad K. (2014). Competence and regulatory interactions during regeneration in plants. Front. Plant Sci..

[26] Podwyszyńska M., Marasek-Ciolakowska A. (2020). Micropropagation of tulip via somatic embryogenesis. Agronomy.

[27] Ge X., Fan G., Chai L., Guo W. (2010). Cloning, molecular characterization and expression analysis of a *SOMATIC EMBRYOGENESIS RECEPTOR-LIKE KINASE* gene (*CitSERK1-like*) in Valencia sweet orange. Acta Physiol. Plant..

[28] Avila-Victor C.M., Arjona-Suárez E.J., Iracheta-Donjuan L., Valdez-Carrasco J.M., Gómez-Merino F.C., Robledo-Paz A. (2023). Callus type, growth regulators, and phytagel on indirect somatic embryogenesis of coffee (*Coffea arabica* L. var. Colombia). Plants.

[29] Campos N.A., Panis B., Carpentier S.C. (2017). Somatic embryogenesis in coffee: The evolution of biotechnology and the integration of omics technologies offer great opportunities. Front. Plant Sci..

[30] Verdeil J.L., Alemanno L., Niemenak N., Tranbarger T.J. (2007). Pluripotent versus totipotent plant stem cells: Dependence versus autonomy?. Trends Plant Sci..

[31] Delporte F., Pretova A., du Jardin P., Watillon B. (2014). Morpho-histology and genotype dependence of in vitro morphogenesis in mature embryo cultures of wheat. Protoplasma.

[32] Zhou X., Zheng R., Liu G., Xu Y., Zhou Y., Laux T., Zhen Y., Harding S.A., Shi J., Chen J. (2017). Desiccation treatment and endogenous IAA levels are key factors influencing high frequency somatic embryogenesis in *Cunninghamia lanceolata* (Lamb.) Hook. Front. Plant Sci..

[33] Venkataiah P., Bhanuprakash P., Suman Kalyan S., Subhash K. (2016). Somatic embryogenesis and plant regeneration of *Capsicum baccatum* L. J. Genet. Eng. Biotechnol..

[34] Singh R., Rai M.K., Kumari N. (2015). Somatic embryogenesis and plant regeneration in *Sapindus mukorossi* Gaertn. from leaf-derived callus induced with 6-Benzylaminopurine. Appl. Biochem. Biotechnol..

[35] Azizi-Dargahlou S., Pouresmaeil M. (2024). *Agrobacterium tumefaciens*-Mediated Plant Transformation: A Review. Mol. Biotechnol..

[36] Wang X., Chen S., Zhang H., Luo P., Zhou F., Zeng B., Xu J., Fan C. (2022). *Agrobacterium*-mediated genetic transformation of the most widely cultivated superior clone *Eucalyptus urophylla* × *Eucalyptus grandis* DH32-29 in Southern China. Front. Plant Sci..

[37] Jadhav M.P., Katageri I.S. (2017). *Agrobacterium tumefaciens* mediated genetic transformation in coker-312 (*Gossypium hirsutum* L.) using hypocotyls explants. Int. J. Curr. Microbiol. App. Sci..

[38] Zhang X., Wu Q., Lin S., Zhang Z., Wang Z., Wang Q., Yan X., Bendahmane M., Bao M., Fu X. (2021). Regeneration and *Agrobacterium*-mediated genetic transformation in *Dianthus chinensis*. Sci. Hortic..

[39] Han J.L., Wang H., Ye H.C., Liu Y., Li Z.Q., Zhang Y., Zhang Y.S., Yan F., Li G.F. (2005). High efficiency of genetic transformation and regeneration of *Artemisia annua* L. via *Agrobacterium tumefaciens*-mediated procedure. Plant Sci..

[40] Zhang B., Zhao X., Zhang L., Liu H., Zhu C., Ma Z. (2022). *Agrobacterium tumefaciens* mediated genetic transformation of *Tripterygium wilfordii* and its application to enhance the accumulation of triptolide. Ind. Crop. Prod..

[41] Li X., Jiang Z., Shen Y., Li F., Yu X., Qu S. (2018). In vitro regeneration and *Agrobacterium tumefaciens*-mediated genetic transformation of *D. lotus* (*Diospyros lotus* L.). Sci. Hortic..

[42] He Y., Zhang T., Sun H., Zhan H., Zhao Y. (2020). A reporter for noninvasively monitoring gene expression and plant transformation. Hortic. Res..

[43] Zhang J., Yu D., Zhang Y., Liu K., Xu K., Zhang F., Wang J., Tan G., Nie X., Ji Q. (2017). Vacuum and co-cultivation agroinfiltration of (Germinated) seeds results in Tobacco Rattle Virus (TRV) mediated whole-plant Virus-Induced Gene Silencing (VIGS) in wheat and maize. Front. Plant Sci..

[44] Yang Q., Fan Y., Luo S., Liu C., Yuan S. (2024). Virus-induced gene silencing (VIGS) in *Hydrangea macrophylla* and functional analysis of *HmF3*′*5*′*H*. Plants.

[45] Livak K.J., Schmittgen T.D. (2001). Analysis of relative gene expression data using real-time quantitative PCR and the 2^−ΔΔCT^ method. Methods.

