# Supplementary material for: Somatic Embryogenesis and Genetic Transformation of Caragana intermedia
Source: Plants (Basel). 2025 May 21;14(10):1545. doi: 10.3390/plants14101545 (PMC12114626; doi:10.3390/plants14101545)
Supplement: Supplementary file 1 [file plants-14-01545-s001.zip › plants-3643794-supplementary.pdf]

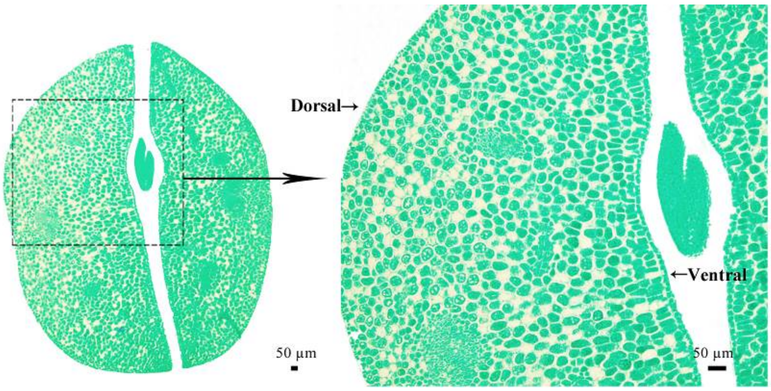

Supplementary Figure S1. Cotyledon section

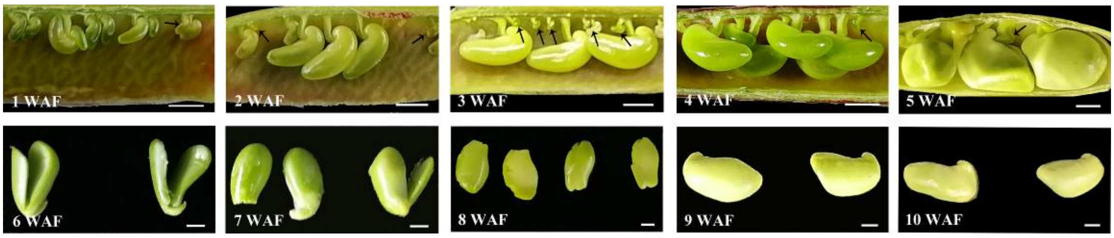

Supplementary Figure S2. Morphological changes in *C. intermedia* seeds during development
The arrows showed ovules aborting without fertilization. Bar: 2000 μm

Supplementary Table S1. Effects of the modes of cotyledon contacting medium on cotyledon-stage embryos induction

Contacting modes	Cotyledon-stage embryo induction rate (%)	Number of cotyledon-stage embryos
The ventral side	56.50±5.45	11.11±1.21
The dorsal side	36.90±5.15	8.45±1.83

Note: Mean values of three independent experiments (±) with standard errors. The same below.

Supplementary Table S2. Germination of cotyledon-stage embryos

Culture medium	Germination rate (%)	Growth state
1/2 MS + 0.050mg/L NAA	0	Cotyledon-stage embryos produced callus and subsequently exhibited browning
1/2 MS + 0.010mg/L NAA	0	
1/2 MS + 0.005mg/L NAA	0	
1/2 MS	0	Cotyledon-stage embryos gradually browned
MS	0	Cotyledon-stage embryos gradually browned
HS-MS	34.36±5.41	Germination plantlets grew normally
1/4 MS	5.34±0.98	Cotyledon-stage embryos gradually browned

Supplementary Table S3. Basic medium components

Component type	Reagent name	Composition content (mg/L)			
		MS	1/2 MS	HS-MS	1/4 MS
Macronutrient salt base	NH_4NO_3	1650	825	825	412.5
	KNO_3	1900	950	950	475
	$MgSO_4 \cdot 7H_2O$	370	185	185	92.5
	KH_2PO_4	170	85	85	42.5
Microelements	$MnSO_4 \cdot H_2O$	16.9	16.9	8.45	4.225
	$ZnSO_4 \cdot 7H_2O$	8.6	8.6	4.3	2.15
	H_3BO_3	6.2	6.2	3.1	1.55
	KI	0.83	0.83	0.415	0.2075
	$Na_2MoO_4 \cdot 2H_2O$	0.25	0.25	0.125	0.0625
	$CuSO_4 \cdot 5H_2O$	0.03	0.03	0.015	0.0075
	$CoCl_2 \cdot 6H_2O$	0.03	0.03	0.015	0.0075
Organic salts	Glycine	2	2	1	0.5
	VB1	1	1	0.5	0.25
	VB6	0.5	0.5	0.25	0.125
	Niacin	0.5	0.5	0.25	0.125
	Inositol	100	100	50	25
Calcium salt	$CaCl_2 \cdot 2H_2O$	440	220	220	110
Iron salts	$FeSO_4 \cdot 7H_2O$	27.8	27.8	13.9	6.95
	Na_2-EDTA	37.3	37.3	18.65	9.325

Supplementary Table S4. LB basic medium components

Component type	Composition content (g/L)	
	Fluid medium	Solid medium
Tryptone powder	10	10
Yeast extract	5	5
NaCl	10	10
Agar	—	15

Supplementary Table S5. Primer sequences used in this study

Primer name	Primer usage	Sequence (5'-3')
pDR5-*RUBY*-F	Transgenic material identification	CGACGGCACCTACCACTACAAT
pDR5-*RUBY*-R	Transgenic material identification	TTATGGTTCTCTGGGAAGCCTG
pTRV2-*CiPDS*-F	Real-time PCR	TGGGGCTTACCCTAATGTGC
pTRV2-*CiPDS*-R	Real-time PCR	CACCTCATCAGTTACCCGTTCA
qCiEF1α-F	Real-time PCR	CAAAAAGTCCCCTCGTTGTCTC
qCiEF1α-R	Real-time PCR	AGCAATCGTTCTTCCTAATGATCTAA
